# Characterization of the Age-Related Differences in Porcine Acetabulum and Femoral Head Articular Cartilage

**DOI:** 10.1177/19476035231214724

**Published:** 2023-11-29

**Authors:** Nathan P. Fackler, Ryan P. Donahue, Benjamin J. Bielajew, Arya Amirhekmat, Jerry C. Hu, Kyriacos A. Athanasiou, Dean Wang

**Affiliations:** 1 Department of Orthopaedic Surgery, University of California, Irvine, Orange, CA, USA; 2 Department of Orthopaedic Surgery, University of California, San Diego, La Jolla, CA, USA; 3 Department of Biomedical Engineering, University of California, Irvine, Irvine, CA, USA

**Keywords:** cartilage, age, acetabulum, hip, characterization

## Abstract

**Objective:**

The use of porcine animal models for cartilage injury has increased recently due to their similarity with humans with regard to cartilage thickness, limited intrinsic healing of chondral defects, and joint loading biomechanics. However, variations in the mechanical and biochemical properties of porcine hip articular cartilage among various tissue ages and weightbearing (WB) regions are still unknown. This study’s aim was to characterize the mechanical and biochemical properties of porcine hip articular cartilage across various ages and WB regions.

**Methods:**

Articular cartilage explants were harvested from WB and non-weightbearing (NWB) surfaces of the femoral head and acetabulum of domesticated pigs (*Sus scrofa domesticus*) at fetal (gestational age: 80 days), juvenile (6 months), and adult (2 years) ages. Explants underwent compressive stress-relaxation mechanical testing, biochemical analysis for total collagen and glycosaminoglycan (GAG) content, and histological staining.

**Results:**

Juvenile animals consistently had the highest mechanical properties, with 2.2- to 7.6-time increases in relaxation modulus, 1.3- to 2.3-time increases in instantaneous modulus, and 4.1- to 14.2-time increases in viscosity compared with fetal cartilage. Mechanical properties did not significantly differ between the WB and NWB regions. Collagen content was highest in the NWB regions of the juvenile acetabulum (65.3%/dry weight [DW]) and femoral head (75.4%/DW) cartilages. GAG content was highest in the WB region of the juvenile acetabulum (23.7%/DW) and the WB region of the fetal femoral head (27.5%/DW) cartilages. Histological staining for GAG and total collagen content followed the trends from the quantitative biochemical assays.

**Conclusion:**

This study provides a benchmark for the development and validation of preclinical porcine models for hip cartilage pathologies.

## Introduction

Articular cartilage lacks the blood vessels and lymphatic supply necessary for self-repair following injury.^1^ Trauma, overuse, or diseases in cartilage can give rise to osteoarthritis, which is found in 61% to 63% of patients undergoing arthroscopy^2^ and 38% to 47% of individuals over 60 years old in the United States.^3^ This high incidence leads to significant burden on the medical system; therefore, techniques to regenerate and replace articular cartilage, such as microfracture, osteochondral allograft transplantation, and autologous chondrocyte implantation, have been utilized to treat chondral defects. These methods are subject to their own limitations, including donor site morbidity,^4^ limited long-term benefit,^5^ fibrocartilage formation, and restricted applicability for larger defects.^6^ Seeking to address these pitfalls, cartilage repair strategies are continually being investigated to heal articular cartilage injuries.^3,7^

Biochemically, the 2 most abundant components of cartilage extracellular matrix, besides water, are collagen and glycosaminoglycans (GAGs), which make up approximately 50% to 75% and 15% to 30% of the dry weight (DW) of articular cartilage, respectively.^8^ Type II collagen makes up 90% to 95% of articular cartilage collagen content and forms fibrils intertwined with GAGs that contribute to the tensile and compressive stiffness and strength of articular cartilage.^1,9^ Through characterization studies, it is known that collagen and GAG content, compressive strength, and genetic expression within articular cartilage vary with factors such as age,^10^ location in the body,^11^ and weightbearing (WB) region.^12^ Before new cartilage repair strategies can be implemented clinically, preclinical testing in animal models helps to characterize the safety and efficacy of the treatment. When considering cartilage pathology and repair, large animal models have been shown to have thicker articular cartilage and earlier closure of epiphyseal plates, making them a more favorable model of human pathology compared with the thinner, highly vascularized cartilage of small animals such as rodents.^13,14^ Importantly, large animal models are closer in weight to humans, thus mimicking the stresses and biomechanics that would be seen in the human condition. Large animal models, including horse, sheep, goat, and pig, have been integral to the understanding in areas such as osteoarthritis,^15^ ligamentous injury,^16^ and fracture healing.^17^

In particular, the use of the porcine model for the study of cartilage tissue engineering and biomechanics has increased in recent years.^18^ Porcine joint size, cartilage thickness, bone apposition rate, and trabecular thickness mimic the human condition closer than all small animal models and some large animal models, such as dogs.^19^ This homology to human anatomy, along with other factors, such as availability through closed research herds^18^ and well-established surgical technique,^20^ likely contributes to the increasing use of the porcine model in preclinical research. In addition, tissue engineering strategies mimicking development, such as the self-assembling process,^21^ will be informed by characterization and analysis of the structure-function relationships across all age groups from fetal to mature tissues.^21^ In humans, cartilage injuries most commonly occur in the WB knee and hip joints. Although the porcine stifle (knee) is frequently used as a validated cartilage injury model,^22,23^ large animal models for hip cartilage pathologies remain scarce and have not been well characterized for the porcine species. Thus, the purpose of this study is to characterize the biochemical and mechanical properties of both WB and non-weightbearing (NWB) articular cartilage in fetal, juvenile, and adult porcine hips. The hypothesis of this work is that the mechanical and biochemical properties of cartilage will vary with age and between the WB and NWB regions of acetabulum and femoral head cartilages.

## Methods

### Tissue Procurement and Dissection

**Figure 1.**
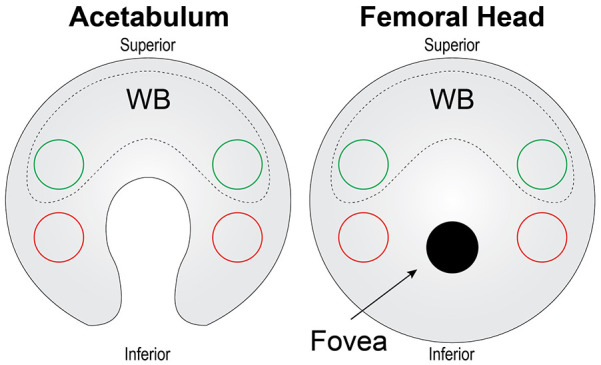
Schematic of tissue harvest for acetabulum and femoral head cartilages. For the acetabulum and femoral head, 2 punches (green circles) from WB cartilage (dashed region) were taken from the superior side of the cartilage surface, while 2 punches (red circles) for NWB cartilage were taken from the inferior surfaces. NWB = non-weightbearing; WB = weightbearing.

Hip joints from domesticated pigs (*Sus scrofa domesticus*, Yorkshire cross, female and male) were obtained from a local abattoir (Corona Cattle Inc., Corona, CA) or an anatomic specimen provider (Nebraska Scientific, Omaha, NE), and therefore, tissues were exempt from approval via an Institutional Animal Care and Use Committee (IACUC). Fresh-frozen whole fetal pigs were purchased from Nebraska Scientific. According to the provided growth chart by the vendor, the fetal pigs were 80 days gestational age. Hip joints from juvenile (5-6 months old) and mature (2-3 years old) pigs, culled for purposes unrelated to this research, were purchased from Corona Cattle Inc. For fetal pigs, unilateral (only the right) hip joints were used, and for juvenile and mature pigs, bilateral hip joints were used. Based on a power analysis using the 20% relaxation modulus from a previous study,^21^ 8 joints per group (*n* = 8) were used here. The hip joint was trimmed using an oscillating saw to cut the femur and pelvis. Upon separation, excess soft tissue was trimmed and the joint frozen *en bloc* for downstream analysis. Upon thawing, joints were opened, and the cartilage of the acetabulum and femoral head were exposed. Cartilage surfaces were visually inspected for signs of osteoarthritic changes, such as chondral defects, osteophytes, and fibrillation prior to further dissection. No gross signs of osteoarthritic change were seen in any of the samples used for testing. The WB and NWB regions of the femoral head and acetabulum were identified using previously validated models of porcine hip contact mechanics.^24^ Two 3-mm-diameter biopsy punches were obtained from the WB and NWB regions (**Fig. 1**) of each cartilage surface from each animal. From each set of two 3-mm-diameter cartilage pieces, 1 piece was used for mechanical testing, and 1 piece was bisected with one half used for biochemical testing and the other half used for histology. For the mechanical and biochemical analyses, the bone was removed from the samples, and all samples were kept at 4°C in phosphate-buffered saline up to 24 hours until downstream analysis.

### Mechanical Testing

Punches of 2-mm-diameter were taken from the 3-mm-diameter, full-thickness cartilage pieces for compressive stress-relaxation testing. The method consisted of height detection using a 0.1 N load followed by an unconfined stress-relaxation algorithm. The height of samples used for mechanical testing was 0.854 ± 0.192 mm. As previously described,^21,25^ the test consisted of 15 cycles of 5% strain with a ramp of 10% strain per second to remove hysteresis followed by a 10% and 20% strain step with a ramp of 10% strain per second until complete relaxation (600 and 900 s, respectively). The resulting stress-relaxation curves were fitted using a standard linear solid model to obtain a relaxation modulus (*E_r_*), an instantaneous modulus (*E_i_*), and coefficient of viscosity (µ) for each strain level in MATLAB. Briefly, the standard linear solid model consists of a spring (*E*_1_) and a dashpot (η) in series that are in parallel with another spring (E_2_). This yields the following constitutive equation, which describes stress (σ) and strain (ε) in the model^26^:

$$\left(E_{1}+E_{2}\right)\frac{d\varepsilon }{dt}+\frac{E_{1}E_{2}}{\eta }\varepsilon =\frac{E_{1}}{\eta }\sigma +\frac{d\sigma }{dt}$$

The relaxation modulus, instantaneous modulus, and coefficient of viscosity are related to these 3 parameters through the following equations^27^:

$$E_{r}=E_{2}$$

$$E_{i}=E_{1}+E_{2}$$

μ=η

### Biochemical Testing

Cartilage pieces were lyophilized for at least 72 hours. Subsequently, a DW was measured. After, the tissue was digested in phosphate-buffered papain solution. A modified hydroxyproline assay was used to measure total collagen content, as previously described.^28^ A Blyscan Biocolor GAG kit was used per the manufacturer’s protocol to measure the GAG content. The values reported were normalized to DW.

### Histology

Cartilage pieces were fixed in 10% formalin for at least 72 hours. Samples were subsequently decalcified using excess 20% disodium ethylenediaminetetraacetic acid for 4 weeks with regular changes every 3 to 4 days. Samples were then processed, embedded, and sectioned at 5 μm. Each section was mounted on a slide and subsequently stained with hematoxylin and eosin (H&E), picrosirius red (Picro), and safranin O with Fast Green counterstain (Saf O). Cell counts were taken from 3 random regions of interest (ROI) measuring 140 µm x 140 µm on a representative H&E section and were reported as the number of cells per mm^2^.

### Statistical Analysis

All statistical analyses were performed in GraphPad Prism 9. Data were analyzed with 2-way analyses of variance (ANOVAs) with *post hoc* Tukey’s honestly significant difference (HSD) test at a significance level of α = .05. A connecting letters report is used to report statistical significance; bars/groups not sharing the same letters are statistically different from each other. All graphs show mean ± standard deviation.

## Results

### Mechanical Testing

**Figure 2.**
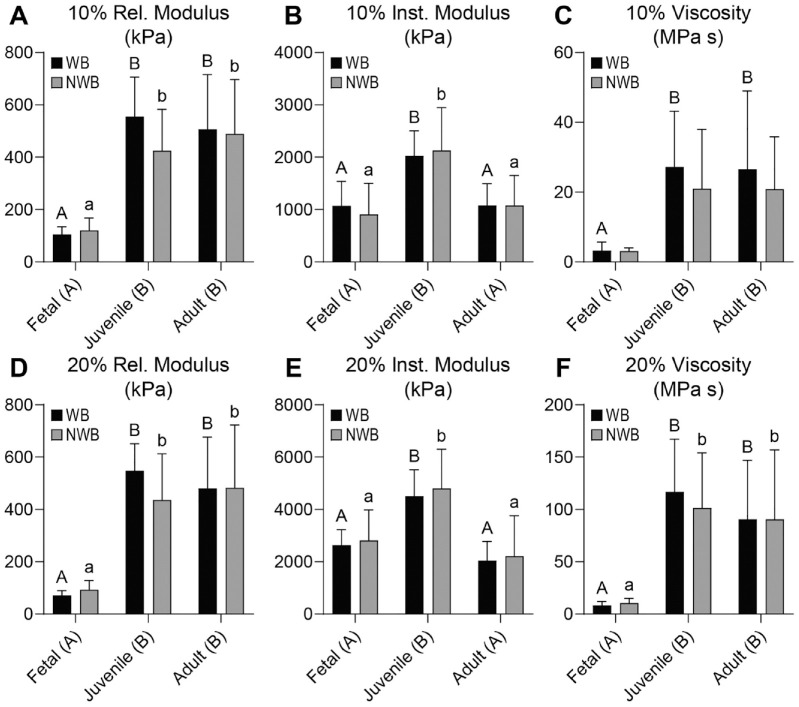
Mechanical properties for acetabulum cartilage. For the acetabulum, (**A**) 10% and (**D**) 20% relaxation moduli trend with age, increasing from fetal to juvenile and adult. For both (**B**) 10% and (**E**) 20% instantaneous modulus, the values peak in the juvenile age group being statistically greater than both fetal and adult groups. Both (**C**) 10% and (**F**) 20% viscosity values follow a similar trend as relaxation modulus, increasing with age. Rel = relaxation; kPa = kilopascal; WB = weightbearing; NWB = non-weightbearing; Inst = instantaneous; MPa s = megapascal-second.

**Figure 3.**
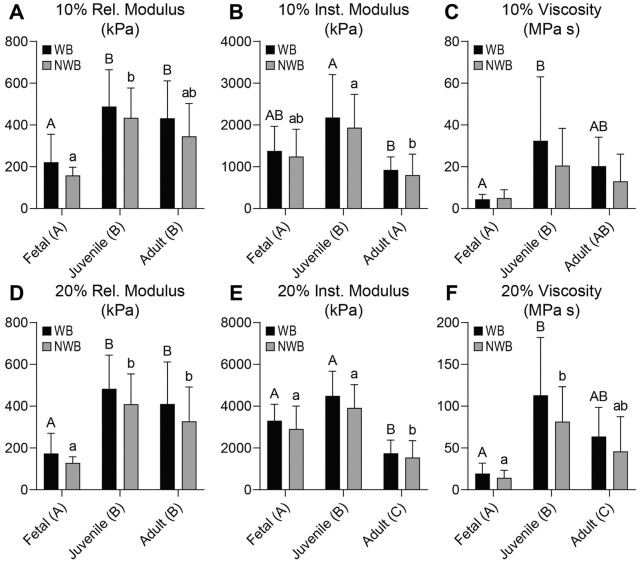
Mechanical properties for femoral head cartilage. For the femoral head, (**A**) 10% and (**D**) 20% relaxation moduli increase with age. For both (**B**) 10% and (**E**) 20% instantaneous modulus, the adult group is significantly lower than the juvenile group. Both (**C**) 10% and (**F**) 20% viscosity values increase from fetal to juvenile groups. Rel = relaxation; kPa = kilopascal; WB = weightbearing; NWB = non-weightbearing; Inst = instantaneous; MPa s = megapascal-second.

In both the acetabulum and femoral head, the 10% and 20% relaxation moduli (*E_r_*) were 2.0 to 6.0 times higher in the juvenile and adult tissues than the fetal tissues (*P* ≤ 0.0027), but there was no difference between the WB and NWB regions in any of the measurements (**Figs. 2A** and **D** Figure 2 and **3A** and **D**). The 10% *E_r_* was 104 to 120 kPa in the fetal acetabulum and 425 to 555 kPa in the juvenile and adult acetabulum (**Fig. 2A**). The 20% *E_r_* of the acetabulum ranged from 72 to 93 kPa in the fetal tissues to 436 to 548 kPa in the juvenile and adult tissues (**Fig. 2D**). In the femoral head, the fetal tissue had a 10% *E_r_* of 158 to 222 kPa, and the adult and juvenile tissues were 346 to 488 kPa (**Fig. 3A**).

In the acetabulum, the 10% and 20% instantaneous moduli (*E_i_*) were 1.7 to 2.2 times higher in the juvenile tissue than fetal or adult tissues (*P* ≤ 0.0003) (**Fig. 2B** and **E**). In the femoral head, the 10% and 20% *E_i_* were 1.4 to 2.6 times higher in the juvenile group than fetal or adult groups (*P* ≤ 0.0169) (**Fig. 3B** and **E**). Differences between 10% and 20% *E_i_* for WB and NWB regions of both the acetabulum and femoral head were not significant. With respect to ranges, in the acetabulum, the 10% *E_i_* ranged from 907 to 1080 kPa in the fetal and adult cartilage to 2028 to 2127 kPa in the juvenile cartilage (**Fig. 2B**). For 20% *E_i_*, fetal and adult acetabulum tissue had values of 2045 to 2815 kPa, while the juvenile tissues yielded values of 4509 to 4806 kPa (**Fig. 2E**). In the femoral head, the 10% *E_i_* ranged between 799 and 1380 kPa in the fetal and adult cartilages, and 1936 to 2177 kPa in the juvenile cartilage (**Fig. 3B**). The 20% *E_i_* exhibited values in the range of 1544 to 1745 kPa for adult, 2909 to 3302 kPa for fetal, and 3919 to 4495 kPa for juvenile femoral head cartilages (**Fig. 3E**).

In the acetabulum, the 10% and 20% viscosities (η) were 7.4 to 11.6 times higher in the juvenile and adult cartilages than the fetal tissue (*P* ≤ 0.0016), with no significant effect between the WB and NWB regions (**Fig. 2C** and **F**). In the femoral head, the juvenile cartilage had 5.6 times higher 10% η than fetal cartilage (*P* = 0.0044), but adult cartilage was not significantly different from either. For 20% η in the femoral head, juvenile cartilage had 1.8 times higher values than adult tissue (*P* = 0.0163), which was 3.3 times higher than fetal cartilage (*P* = 0.0428).

### Biochemical Testing

**Figure 4.**
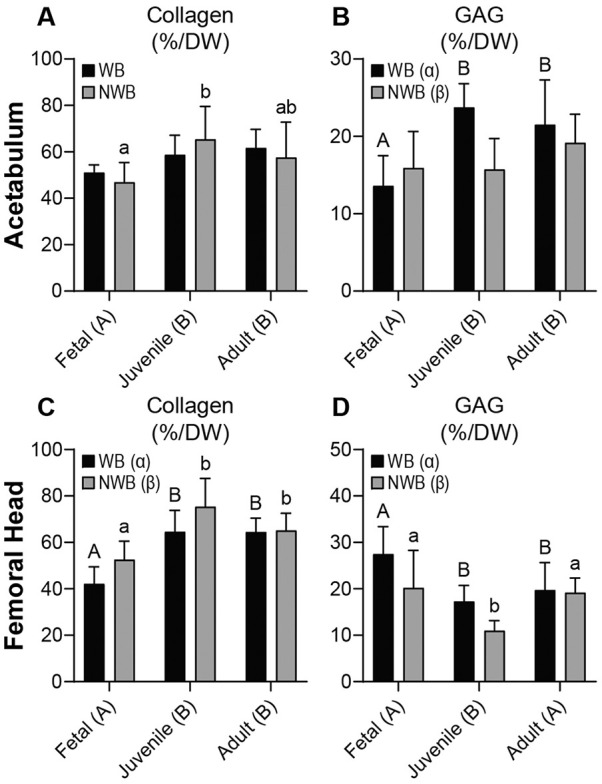
Biochemical content for acetabulum and femoral head cartilages. For the acetabulum, (**A**) collagen content is significantly higher in the juvenile and adult groups compared with fetal tissues, while (**B**) GAG content is significantly higher in the WB regions compared with the NWB regions. For the femoral head, (**C**) collagen content is higher in the juvenile and adult groups, while (**D**) GAG content is significantly lower in the juvenile group. GAG = glycosaminoglycan; WB = weightbearing; NWB = non-weightbearing; DW = dry weight.

Collagen (COL) content of the acetabulum and femoral head cartilages ranged from approximately 42.1% to 75.4% COL/DW (**Fig. 4A** and **C**). In the acetabulum, juvenile and adult cartilages had 1.2 to 1.3 times more COL/DW than fetal cartilage (*P* ≤ 0.0241), and the difference between the WB and NWB regions was not significant (**Fig. 4A**). In the femoral head, the trend among different ages of donors was the same; juvenile and adult cartilages had 1.4 to 1.5 times more COL/DW than fetal (*P* < 0.0001) (**Fig. 4C**). Unlike the acetabulum, the femoral head cartilages in the WB region contained 11% less collagen than the NWB regions (*P* = 0.0065) (**Fig. 4C**).

GAG content was also quantified in WB and NWB regions of the acetabulum and femoral head cartilages. In the acetabulum, juvenile and adult tissues had 1.3 to 1.4 times more GAG/DW than fetal tissue (*P* ≤ 0.0113), and WB regions had 16% more GAG/DW than NWB areas (*P* = 0.0469) (**Fig. 4B**). Out of all tissues tested in the acetabulum, the WB region of the juvenile acetabulum had the highest GAG/DW at 23.7 ± 3.1% (**Fig. 4B**). Unlike the WB region trend, the age of donor cartilage did not significantly change the GAG/DW in the NWB regions of the acetabulum (**Fig. 4B**). The femoral head cartilage showed a similar trend in the WB regions which had more GAG/DW than NWB regions (*P* = 0.0039), but the trend among donor ages was different; juvenile cartilage had 27% to 41% less GAG/DW than fetal or adult (*P* ≤ 0.0178) (**Fig. 4D**).

### Histology

**Figure 5.**
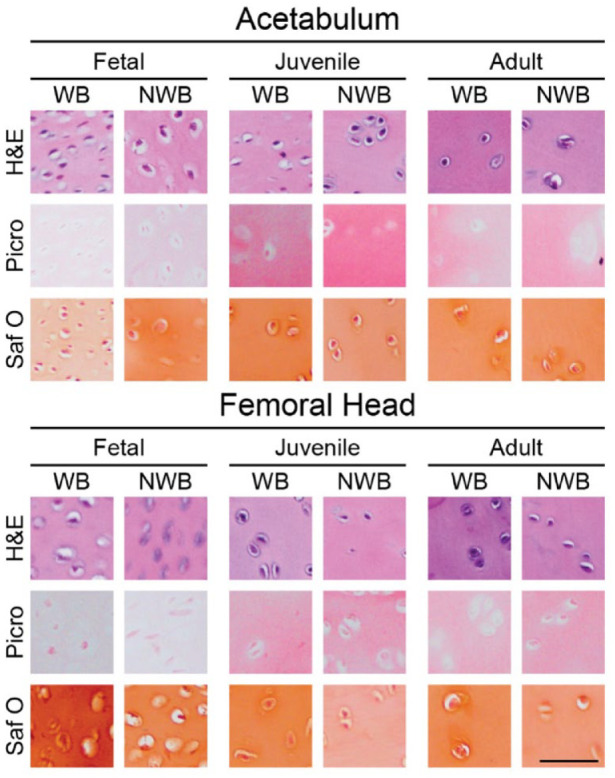
Histology of acetabulum and femoral head cartilages. H&E for general cellular morphology, Picro for collagen content, and Saf O for GAG content are presented. Generally, cellularity becomes less as tissue age. In addition, Picro and Saf O staining intensities follow quantitative biochemical content. Scale bar = 50 μm. H&E = hematoxylin and eosin; Saf O = safranin O with fast green counterstain; GAG = glycosaminoglycan; Picro = picrosirius red; WB = weightbearing; NWB = non-weightbearing.

H&E, Picro, and Saf O histological stains are shown at 20x magnification (**Fig. 5**, Supplementary Figure 1). Cells can be visualized in all 3 staining modalities, where it is generally observed that the fetal cartilage is more cellular than the juvenile and adult cartilages, both in the acetabulum and femoral head (**Fig. 5**). For H&E, the background eosin staining remains relatively consistent throughout all samples (**Fig. 5**). Shown in the quantitative biochemical analysis is that the fetal cartilage contained the least COL/DW in both the acetabulum and femoral head (**Fig. 4A** and **C**), which is reflected in the Picro staining; the red-stained collagen is much more intense in the juvenile and adult sections (**Fig. 5**). For Saf O, the most intense GAG staining is shown in the WB region of fetal femoral head cartilage, which is reflected in the quantitative biochemical analysis (**Figs. 4B** and **D** and **5**). Fetal cartilage was significantly more cellular than juvenile (*P* < 0.0001) and adult (*P* < 0.0001) groups in both the acetabulum and femoral head, although no significant differences were found between WB and NWB groups in either location (**Table 1**).

**Table 1.** Cell counts for acetabulum and femoral head cartilages.

	Acetabulum			Femoral Head		
	Fetal (A)	Juvenile (B)	Adult (C)	Fetal (A)	Juvenile (B)	Adult (B)
WB	2,194 ± 88^a^	799 ± 164^b^	391 ± 29^c^	2,177 ± 490^a^	867 ± 102^b^	697 ± 147^b^
NWB	1,718 ± 281^a^	867 ± 222^b^	340 ± 59^c^	2,143 ± 153^a^	1,037 ± 179^b^	595 ± 78^b^

Numbers reported in cells per mm^2^. In the acetabulum, fetal tissue was significantly more cellular than juvenile and adult. In addition, juvenile acetabulum cartilage was significantly more cellular than adult tissue. In the femoral head, fetal tissue was significantly more cellular than juvenile and adult tissue. Differences between juvenile and adult tissue cellularity in the femoral head were not significant.

WB = weightbearing; NWB = non-weightbearing.

## Discussion

Cartilage repair treatments of articular defects in the hip has been increasing in recent years,^3,7^ making the characterization of preclinical animal models even more critical. Specific to the objective of this study, the porcine model for articular cartilage injury has been gaining popularity due to its homology to human anatomy, availability through controlled herds, and well-established surgical techniques.^20^ However, the articular cartilage of the porcine hip has not been characterized to date. This study aimed to characterize the mechanical and biochemical properties of porcine articular cartilage across various ages and WB regions of the acetabulum and femoral head. The hypothesis that there would be age-dependent changes in mechanical properties and collagen content was confirmed. However, age-dependent changes in GAG content were less clear. Across all ages, juvenile animals consistently had the highest mechanical properties, with 2.2- to 7.6-time increases in relaxation modulus, 1.3- to 2.3-time increases in instantaneous modulus, and 4.1- to 14.2-time increases in viscosity compared with fetal cartilage. Mechanical properties did not change between the WB and NWB regions. Collagen content was highest in the NWB regions of the juvenile acetabulum (65.3%/dry weight [DW]) and femoral head (75.4%/DW) cartilages. GAG content was highest in the WB region of the juvenile acetabulum (23.7%/DW) and the WB region of the fetal femoral head (27.5%/DW) cartilages.

In this study, collagen content appeared to increase with age, with the lowest amount of collagen found in the fetal cartilage and highest in the juvenile and adult cartilage. Other characterization studies have established that the biochemical properties of articular cartilage change as animals age.^10,18,29,30^ In many studies, collagen consistently demonstrates an increase in content with age regardless of the size of the animal or species being studied.^31-33^ However, age-related changes in the GAG content of articular cartilage are less clear and appear to be species-specific. Human and porcine studies have shown GAG content to be highest in fetal cartilage, decreasing with age.^34,35^ Other large animals, such as bovine, have demonstrated a more consistent concentration of GAGs throughout the lifecycle.^33^ The results of this study agree with previously established trends in porcine cartilage, with GAG content in the fetal femoral head being higher than juvenile and adult tissues and collagen content being higher in juvenile and adult compared with fetal. However, deviations from the norm such as low GAG content in the fetal acetabulum were demonstrated. Additional techniques, for example, using fluorescent-assisted carbohydrate electrophoresis (FACE) for GAG subtyping^36^ and mass spectrometry for collagen subtyping,^37^ would help to further investigate the differences between the development of acetabulum and femoral head cartilages in the porcine model.

Similar to other characterization studies, this study found a trend between collagen content and mechanical properties of cartilage, with the fetal groups demonstrating a significantly lower viscosity and relaxation modulus than the juvenile and adult groups. Collagen fibrils have been found to demonstrate a fivefold increase in thickness from fetal to adult age.^31^ Increases in the size and density of articular collagen over time have been correlated with the age-related changes in the mechanical properties of cartilage.^10,12,31,33^ In addition, age-related mechanical changes are correlated with increases in the fixed charge density and osmotic pressures generated by interactions between GAGs and collagen fibrils.^10^ While negatively charged GAGs play a significant role in generating the fixed charge density, increases in collagen content have been shown to independently lead to increases in fixed charge density as well as compressive strength of bovine articular cartilage.^33^

Despite having similar biochemical content to juvenile cartilage, the adult cartilage demonstrated a consistently lower instantaneous modulus than juvenile cartilage, regressing to a modulus similar to fetal cartilage. One potential explanation for this difference is that domesticated pigs have been identified as a model for spontaneously occurring osteoarthritis, and that pre-osteoarthritic changes may be present in the mechanical properties of adult articular cartilage before measurable changes in biochemical content can be appreciated.^21,38,39^ In addition, as cartilage ages, acidic keratan sulfate becomes more prevalent in the extracellular matrix, leading to a decrease in fixed charge density within articular cartilage.^10,33,40^ A disproportionate increase in keratan sulfate can drop the fixed charge density of articular cartilage by as much as 50%.^33^ A drop in fixed charge density leads to a decrease in water content of articular cartilage, making it more vulnerable to compressive forces. Furthermore, this change in charge and water content can decrease collagen fibril interconnectivity between cartilage zones, making the superficial zone more susceptible to strain and impact forces.^10^ Therefore, increases in the mechanical properties seen from fetal to juvenile pigs in this study may be driven by changes in collagen content while changes from juvenile to adult may be driven more by an age-related increase in keratan sulfate. Future studies utilizing more specific GAG quantification techniques, such as FACE, will be needed to test this hypothesis.

When separating tissues out into WB and NWB groups, this study found that WB portions of cartilage demonstrated more GAG/DW on average than NWB regions for both acetabulum and femoral head. Previous studies focused on differences between WB and NWB regions of femoral head cartilage have reported higher stiffness and resistance to compression in WB than NWB portions of the femoral head.^41,42^ More recent studies of articular cartilage have attributed this difference in mechanical properties to an increase in GAG content of the extracellular matrix of different regions’ cartilage.^43,44^ Previous studies have found that collagen fibers in WB and NWB regions of cartilage have the same diameter and degree of crosslinking, concluding that the mechanical differences seen in these tissues are likely due in large part to differences in GAG content.^12^ Despite significant differences in GAG content, this study found no significant differences in mechanical properties between WB and NWB cartilage. Importantly, these previous studies used cartilage from different animals and age groups, and therefore, data from this study need to be taken in the context of the specific groups examined (i.e., porcine fetal, juvenile, and adult).

It is important to note in the interpretation of this data that the WB and NWB surfaces identified in this study were harvested using a model of contact mechanics for porcine hip hemiarthroplasty.^24^ Currently, no studies exist examining native contact mechanics for the porcine hip. Tribological studies examining the porcine hip in both native and hemiarthroplasty hips have shown that the area of wear for hemiarthroplasty is more centralized relative to native hips, and therefore, there is high confidence that the hemiarthroplasty model used likely captures the WB portion.^45^ Samples of NWB portions of the femoral head and acetabulum were taken far from the reported WB surfaces in an attempt to decrease incidence of overlapping cartilage, but future studies should interrogate the exact WB and NWB regions of the porcine hip.

Porcine models have shown promise for preclinical studies for cartilage injury, including those focused on engineered articular cartilage. With increased use of this model, characterizations of tissue across multiple variables (i.e. age, location, WB region, etc.) become necessary for development of site-specific neocartilage. The data in this article provide a reference point for the mechanical and biochemical properties of the porcine hip across varying age groups and WB regions. The data presented in this study create an important outline for future investigation of articular cartilage repair utilizing the preclinical porcine model. Studies further characterizing changes in the extracellular matrix throughout fetal development as well as during the transition from juvenile to adult would allow for a deeper understanding of the mechanical changes seen in aging cartilage.

## Supplemental Material

sj-jpg-1-car-10.1177_19476035231214724 – Supplemental material for Characterization of the Age-Related Differences in Porcine Acetabulum and Femoral Head Articular Cartilage Supplemental material, sj-jpg-1-car-10.1177_19476035231214724 for Characterization of the Age-Related Differences in Porcine Acetabulum and Femoral Head Articular Cartilage by Nathan P. Fackler, Ryan P. Donahue, Benjamin J. Bielajew, Arya Amirhekmat, Jerry C. Hu, Kyriacos A. Athanasiou and Dean Wang in CARTILAGE
