# Supplementary figures and images for: Characterization of the Age-Related Differences in Porcine Acetabulum and Femoral Head Articular Cartilage
Source: Cartilage. 2023 Nov 29;16(3):366–75. doi: 10.1177/19476035231214724 (PMC12276429; doi:10.1177/19476035231214724)

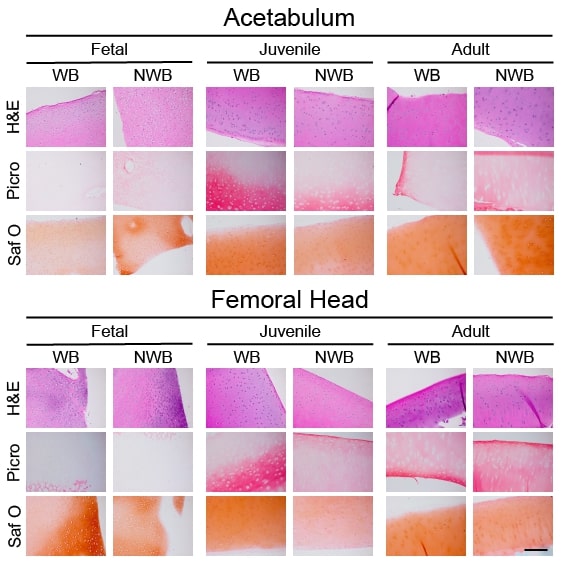

Supplement: sj-jpg-1-car-10.1177_19476035231214724 – Supplemental material for Characterization of the Age-Related Differences in Porcine Acetabulum and Femoral Head Articular Cartilage [file sj-jpg-1-car-10.1177_19476035231214724.jpg]
